# Use of a Deformable Atlas to Identify Cryptic Critical Structures in the Treatment of Glioblastoma Multiforme

**DOI:** 10.1371/journal.pone.0032098

**Published:** 2012-03-26

**Authors:** David C. Weksberg, Stephen D. Bilton, Eric L. Chang

**Affiliations:** 1 MD Anderson Cancer Center, Department of Radiation Oncology, University of Texas, Houston, Texas, United States of America; 2 Department of Radiation Oncology, University of Southern California Keck School of Medicine, Norris Cancer Hospital, Los Angeles, California, United States of America; NIH, United States of America

## Abstract

Dose constraints for traditional neural critical structures (e.g. optic chiasm, brain stem) are a standard component of planning radiation therapy to the central nervous system. Increasingly, investigators are becoming interested in accounting for the dose delivered to other non-target neural structures (e.g. hippocampi), which are not easily identified on axial imaging. In this pilot study, a commercially available digital atlas was used to identify cryptic neural structures (hippocampus, optic radiations, and visual cortices) in 6 patients who received intensity modulated radiation therapy (IMRT) as part of multimodal management of glioblastoma multiforme (GBM). The patient's original IMRT plans were re-optimized, with avoidance parameters for the newly identified critical structures. Re-optimization was able to reduce both mean and maximum dose to the volumes of interest, with a more pronounced effect for contralateral structures. Mean dose was reduced by 11% and 3% to contralateral and ipsilateral structures, respectively, with comparable reduction in maximum dose of 10% and 2%, respectively. Importantly, target coverage was not compromised, with an average change in coverage of 0.2%. Overall, our results demonstrate the feasibility of incorporating tools for cryptic critical structure identification into the treatment planning process for GBM.

## Introduction

Malignant gliomas represent the most common primary central nervous system (CNS) malignancy in adults, with anaplastic astrocytoma (AA) and glioblastoma multiforme (GBM) accounting for 60% of malignant CNS gliomas [1]. Radiation therapy is a cornerstone of treatment for these neoplasms, and, owing to the diffusely infiltrative nature of these tumors, large treatment volumes are often required to deliver optimal treatment. While the prognosis for high-grade glioma generally remains poor, with the advent of concurrent temozolamide (TMZ) therapy [2], [3], [4], and the identification of 1p19q loss of heterozygosity [5], [6], [7] and *IDH1* mutations [8] as favorable prognostic indicators, there are now subsets of patients for whom the median survival is predicted to be measured in years. As the ability to identify these patients with favorable responses improves, minimizing iatrogenic neurotoxicity will be of growing importance.

Planning radiation treatment to the CNS involves the delineation of critical organ at risk structures, such as the optic chiasm, brainstem, and cochlea, in order to reduce the risk of treatment toxicity. However, while structures such as the chiasm are readily identifiable on axial imaging, other structures at potential risk, such as the optic radiations, hippocampi, or visual cortices, are not readily segmented using conventional planning systems. Thus, we sought to demonstrate the feasibility of using a deformable anatomic digital atlas as a tool to aid in identifying and contouring these cryptic neural structures. Here we present the results of a pilot studying applying this technique to six patients who underwent radiation treatment for GBM.

## Materials and Methods

### Patient characteristics

After obtaining institutional IRB approval for retrospective dosimetric analysis, the treatment plans of 6 patients treated for high-grade glioma at the University of Texas MD Anderson Cancer Center between 2008 and 2010 were selected for analysis. 5 patients were treated for primary disease, and 1 patient was treated for recurrent disease. Patient characteristics are summarized in Table 1. No external funding was received for this study.

**Table 1:** Patient characteristics.

Pt	Age	Gender	Tumor type	Location	GTV	PTV
1	63	M	GBM (recurrent)	R temporal lobe	123 cc	185 cc
2	50	F	GBM	R frontal lobe	17 cc	251 cc
3	56	M	GBM	R frontal lobe	80 cc	564 cc
4	60	F	GBM	R basal ganglia	19 cc	297cc
5	62	F	GBM	R temporal lobe	101 cc	482 cc
6	62	F	GBM	L parietal lobe	38 cc	454 cc

### Treatment Planning and Target Volumes

**Figure 1:**
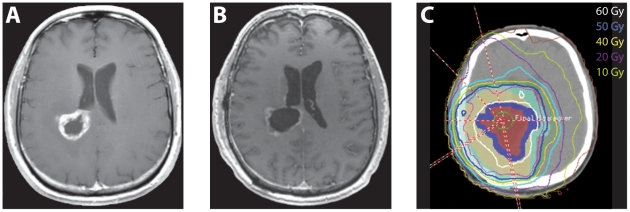
Diagnostic imaging and treatment plan from a representative case. A) Pre-operative contrast enhanced MRI. B) Post-operative contrast enhanced MRI showing resection cavity. C) Treatment planning CT with isodose lines depicting the dose distribution.

Pinnacle treatment planning software (Version 9) with Direct Machine Parameter Optimization (DMPO) was used for treatment planning. For patients with primary tumors, the patient's pre-operative contrast-enhanced MRI was registered with the treatment planning CT scan obtained at the time of simulation, to aid in delineation of the clinical target volume (CTV). A high-dose CTV (CTV1) was identified, comprising the surgical cavity, as well as the areas of contrast enhancement and areas of FLAIR signal abnormality deriving from tumor infiltration. CTV1 was uniformly expanded by 5 mm to account for set-up uncertainty – this expansion created a planning target volume (PTV), termed “Boost PTV,” which was planned to receive 60 Gy in 30 fractions. To create the low-dose CTV (CTV2), CTV1 was uniformly expanded by 2 cm, and contours were then edited to exclude air, bone, and brain parenchyma protected by anatomic barriers. A 5 mm uniform expansion for set-up uncertainty was performed, with the resultant volume “PTV50” receiving 50 Gy in 30 fractions. Intensity modulated radiation therapy (IMRT) treatment plans were designed to deliver a simultaneous integrated boost (2 Gy per fraction to “Boost PTV” and ∼1.66 Gy per fraction to “PTV50”). A representative treatment plan is illustrated in Figure 1. For the 1 patient with recurrent disease (and a history of prior radiation for his primary disease), gross recurrent disease was contoured, and expanded 5 mm for set-up uncertainty – the resultant PTV was planned for 40 Gy in 20 fractions.

### Organ at risk (OAR) segmentation

**Figure 2:**
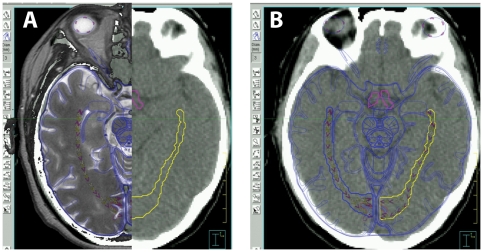
Anatom-E digital atlas. For structure delineation, the display of treatment planning system (Pinnacle) is mirrored to a second computer monitor, and the Anatom-E images are superimposed (A) and adjusted and scaled to achieve registration with the planning CT images. Anatom-E software controls allow blending (B) of the MRI and wireframe views for ease of registration and structure identification.

**Figure 3:**
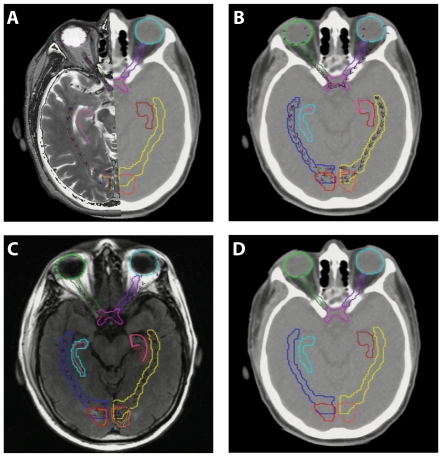
Identification of cryptic critical structures. In A) the overlay of the Anatom-E atlas and planning CT is illustrated. In B), the atlas image has been turned off, leaving the structures alone superimposed on the planning CT. Panel C shows the overlay with the pre-treatment MRI, and Panel D depicts the atlas images turned off, with the resultant contours on the planning CT.

Anatom-E (Houston, TX) is a commercially available deformable anatomic digital atlas [9] which provides axial MRI imaging with functional annotation of CNS structures validated with intraoperative neurosurgical data (J. Pagani, personal communication). We used the Anatom-E atlas to aid in delineation of the optic radiations, visual cortices, and hippocampi. To accomplish this, the Pinnacle treatment planning display console was mirrored to the Anatom-E display. Software image controls allow blending of MRI images (for ease of registration) and wireframe structural anatomy (for display of functional areas and white matter tracts), as shown in Figure 2. The deformable anatomic atlas was registered to the planning CT and pre-operative MRI images. To accomplish this, the atlas template was manually deformed on a slice by slice basis using an affine (linear) transformation, which allows compensation for variation in brain dimensions and patient head position, with the surfaces of the cerebral hemispheres serving as a landmark. The highly constant central sulcus was used to verify alignment as previously described [10], [11]. Selected structures were contoured based on the atlas guideline (Figure 3). In 3 patients, ipsilateral OAR structures were impacted by resection defects or gross disease: patient 1 – gross recurrent disease abutted ipsilateral OARs with minimal mass effect, patient 4 – the surgical cavity abutted a small section of the optic radiations anteriorly, patient 6 - resected disease prevented the contouring of the ipsilateral optic radiations. Contralateral structures were unaffected in all patients.

IMRT treatments were then re-planned, incorporating the newly identified volumes as avoidance structures using soft constraints designed to reduce OAR dose without compromising tumor coverage. In order to accomplish this, the original beam arrangement and isocenter were preserved, as were the initial optimization parameters. Additional optimization structures for each critical structure were created and set approximately 10–20% lower than the dose delivered by the original plan. Progressive rounds of optimization were performed until the optimization results began to yield plans with inadequate prescription dose coverage (<95% target volume). At this point, OAR objectives were relaxed to restore tumor coverage. Overall, the additional optimization procedures added approximately 2 hours to the planning time for a given patient.

## Results

**Figure 4:**
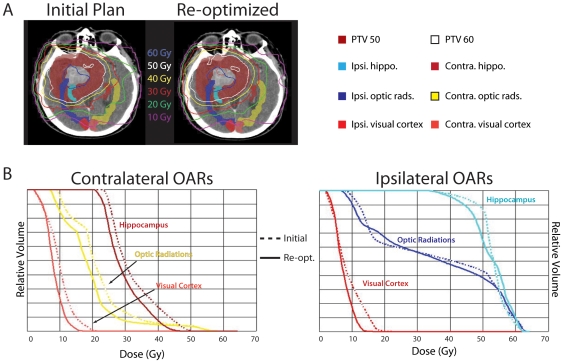
Re-optimized treatment plans reduce dose to cryptic OARs. A) Comparison of isodose lines on an axial slice from a representative patient. B) Dose-volume histogram analysis for the plan depicted in (A).

Re-optimization of the IMRT treatment plans generated new treatment plans that reduced dose to the identified OARs. A representative case is illustrated in Figure 4, with dose-volume histogram analysis (DVH) demonstrating shifts in the dose-volume curves following re-optimization. A qualitative dose-reduction effect was more pronounced for OARs contralateral to the tumor bed.

**Figure 5:**
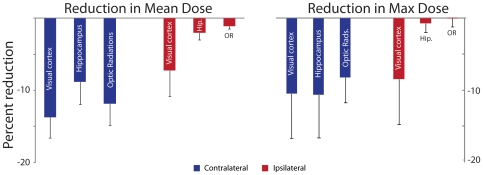
Re-optimization reduces mean and maximum dose parameters. The average percent reduction in mean (left panel) and maximum (right panel) dose is shown for the ipsilateral and contralateral visual cortices, hippocampi (hip.) and optic radiations (OR). Error bars represent the standard error of the mean.

In order to quantify the degree of dose-reduction, we analyzed the change in mean dose and maximum dose to critical structures after re-optimization. With regard to mean dose, an average dose reduction of 3% was achieved for critical structures ipsilateral to the tumor bed (range 0%–16%). The ability to reduce dose to ipsilateral structures was limited by the need to preserve tumor coverage – in many cases, these ipsilateral structures lay within or in immediate proximity to the tumor bed. However, as anticipated, the effect of re-optimization on contralateral structures was more pronounced – an average dose reduction of 11% was achieved for critical structures contralateral to the tumor bed, with 93% of these structures showing some dose reduction (range 0.4% increase to 22% reduction). Generally, the visual cortices were located at the greatest distance from the tumor bed – these structures correspondingly enjoyed the greatest magnitude of dose reduction (Figure 5). Importantly, the goal of critical structure sparing did not compromise tumor coverage – the average change in dose to the PTV was a 0.2% increase (range 0.7% decrease to 0.9% increase). Analysis of reduction in maximum dose to OARs revealed similar results, albeit with somewhat more modest reductions (average reduction of 2.4% to ipsilateral structures and 9.7% to contralateral structures).

## Discussion

Strategies to reduce iatrogenic neurotoxicity secondary to CNS irradiation have been of longstanding interest, and in recent years, growing attention has been given to efforts to assess and avert adverse neurocognitive outcomes in a variety of clinical situations (e.g. dose reduction for patients with primary CNS lymphoma [12], and SRS alone for patients with 1–3 brain metastases [13], [14]).

In addition to dose reduction where appropriate, an additional strategy under active investigation is that of reducing unnecessary dose to functional neural pathways. An ongoing trial administered by the Radiation Therapy Oncology Group (RTOG) – RTOG 0933 – examines the potential benefit of hippocampal avoidance for patients undergoing whole brain radiation therapy (WBRT) for cancers metastatic to the brain. This trial seeks to test whether IMRT techniques that spare the hippocampus will yield improved neurocognitive outcomes. The patient population for this study includes patients with a RTOG brain metastasis recursive partitioning analysis (RPA) class of I–II – translating to a median overall survival of less than one year [15]. The expected median survival of GBM patients, by contrast, ranges from 12–17 months [2], [3] suggesting that efforts to reduce potential neurocognitive effects of radiation are appropriate in this population as well.

Two recent reports also explore the feasibility of accounting for dose to non-traditional OARs in treatment planning for high-grade glioma. As part of an analysis of advanced treatment delivery technologies, a Danish study [16] examined the dose delivered to the hippocampi from IMRT plans compared to treatment planning with inversely optimized rotational therapy (RapidArc) and intensity modulated proton therapy (IMPT). Details on the delineation of the hippocampal OAR were not specified, but the authors found that IMPT plans gave the lowest average dose to the contralateral hippocampus. Treatment planning results in the absence of efforts to spare the hippocampus were not described, precluding quantification of what dose reduction was achieved. Another recent study from Marsh et al [17] examined sparing of the limbic system, hippocampus and neural stem-cell niches in high-grade glioma. This study used more stringent optimization criteria, setting a maximum dose of 8 Gy to the contralateral hippocampus, and reported a more dramatic reduction of dose to the contralateral hippocampus (57% reduction in mean dose). However, this analysis is not directly comparable to the present study, as the inclusion of the posterior optic radiations as avoidance structures in this analysis affects the ability to minimize dose to multiple geographic regions of the brain.

Additionally, while several guidelines for hippocampal sparing have been published in the clinical literature [18], [19], this complex structure still requires expertise for accurate segmentation – as underscored by the fact that the RTOG 0933 trial requires central review of hippocampal OAR contours. Our work demonstrates the feasibility of using a deformable registration tool to accomplish the delineation of this structure – previous dosimetric studies do not provide detailed descriptions of the methods used to identify the hippocampi. Additionally, to our knowledge, this study is the first to include analysis of dose avoidance to the posterior optic pathways, for which there are no consensus contouring guidelines. Owing to the extensive annotation of the digital anatomic atlas, this technique could be extended to include other functional areas as desired.

Ultimately, as data on neurocognitive effects of radiation therapy emerge from ongoing clinical investigations, and knowledge of functional neuroanatomy is further supplemented with improvements in functional imaging, accounting for dose delivered to these circuits will become increasingly important in radiation oncology treatment planning. Our study reduced dose to multiple OARs simultaneously while preserving target coverage. As data regarding the clinical effects of cryptic OAR sparing approaches emerges, treatment planning objectives can be altered accordingly in pursuit of dose reductions of larger magnitude for an individualized clinical scenario.

Future studies will help define the risk-benefit ratio of reduced target coverage in favor of OAR preservation, as well as help establish relative priorities for OAR preservation. Overall, our work illustrates how a deformable anatomic atlas can be used to reduce the dose delivered to cryptic critical structures – critical structures that are not accounted for in conventional treatment planning schema were identified and spared without compromising target coverage.
